# Harnessing desiccation-tolerant cyanobacteria as biosensors for LexA-mediated responses in deep space

**DOI:** 10.3389/fmicb.2026.1842062

**Published:** 2026-07-15

**Authors:** Costanza Maria Martella, Giorgia Di Stefano, Antonio Chirico, Andrea D’Agostino, Marco Maria D’Andrea, Flavio Colini, Federico Iacovelli, Mattia Falconi, Adriano Verna, Jessica Scifo, Alessia Cemmi, Daniela Billi

**Affiliations:** 1 Department of Biology, University of Rome “Tor Vergata,”, Rome, Italy; 2 PhD Program in Cellular and Molecular Biology, University of Rome “Tor Vergata”, Rome, Italy; 3 Bioinformatics Research Unit in Infectious Diseases, National Institute for Infectious Diseases “Lazzaro Spallanzani”, IRCCS, Rome, Italy; 4 ENEA-Nuclear Department, Casaccia Research Center, Rome, Italy

**Keywords:** biosensor, desert cyanobacteria, DNA damage, LexA, microgravity and radiation

## Abstract

**Introduction:**

A LexA-mediated biosensor tailored for space applications was developed leveraging the desert cyanobacterium *Chroococcidiopsis* sp. CCMEE 029 engineered with a transcriptional fusion between an SOS-responsive promoter and a luciferase gene reporter.

**Methods:**

The SOS DNA repair system was investigated by bioinformatics analysis that identified a *lexA* gene in the genome CCMEE 029 encoding a protein sharing key structural features with other cyanobacterial homologs. Consensus LexA-binding motifs upstream genes involved in DNA-damage repair, photosynthesis, and oxidative-stress defense were computationally identified and the DNA binding of CCMEE 029’ LexA was confirmed by molecular modeling and molecular dynamics simulations. The expression of *lexA* and *recA* after treatment for 30 min with 10 mM H_2_O_2_ was evaluated by RT-qPCR. Then Chroococcidiopsis sp. CCMEE 029 was transformed with a plasmid carrying a transcriptional fusion between the upstream region of the *recA* gene containing the LexA-binding motif and a firefly luciferase gene. The biosensor responsiveness was tested in response to DNA-damaging agents, after desiccation as well as under simulated microgravity conditions combined with γ-rays.

**Results:**

Upon the addition of the luciferin substrate, Chroococcidiopsis transformants exposed to DNA-damaging agents (H_2_O_2_, ultra-violet C (UVC), and γ-ray irradiation) emitted a bioluminescent signal. A correlation was detected between increased UVC doses and the onset of detectable DNA damage. The biosensor responsiveness was confirmed under simulated microgravity conditions combined with γ-rays used as a proxy of the space environment. Notably, the biosensor responsiveness was retained after 4.5 months of air-dried storage as demonstrated by signal emission after rehydration and UVC and γ-ray irradiation.

**Discussion:**

Although a more global stress management role for LexA remains to be investigated in Chroococcidiopsis sp. CCMEE 029. The tested biosensor’s feature supports a future integration of the air-dried biosensor into satellites and its in-orbit reactivation for real-time monitoring of the effects of space conditions, thus advancing future space exploration.

## Introduction

Human and robotic exploration of space beyond low Earth orbit (LEO) is entering a new phase with recent advances in constructing the Moon-orbiting Gateway and planned human missions to the Moon as a test bed for future missions to Mars (Fogtman et al., 2023). However, deep space exploration faces major challenges stemming from the synergistic interplay of cosmic radiation and altered gravity, which makes their biological effects complex and not fully understood (Afshinnekoo et al., 2020). Such hurdles not only impair human permanence beyond LEO but affect also the biological components of life support systems (BLSS) that are crucial to enable self-sufficient and Earth-independence of human outposts beyond LEO (Marshall Porterfield et al., 2025).

Cyanobacteria and eukaryotic microalgae are key players in BLSS, since they produce oxygen and food. In addition, they can be used as bio-factories providing a wide array of compounds and their biomass can be used as feedstock and biofertilizer/biostimulant for plants and microorganism-based processes (Mapstone et al., 2022; Verseux et al., 2016). Beginning from the 1960s mostly microalgae, like those belonging to the *Chlorella*, *Chlamydomonas*, and *Euglena* genera, have been exposed to flight conditions on-board the Soviet Korabl-Sputnik and Soyuz, the NASA’s Space Shuttle and the International Space Station (ISS) (Mapstone et al., 2022; Niederwieser et al., 2018). Nevertheless, several knowledge gaps remain in operating reliable bioreactors over long periods (De Micco et al., 2023) and in developing strategies to boost BLSS efficiency using synthetic biology (Onofri et al., 2025). Currently, the effect of ionizing radiation and microgravity on *Limnospira indica* PCC8005, a key component of the European Space Agency’s (ESA) Micro-Ecological Life Support System Alternative (MELiSSA) project, is under investigation both with spaceflight experiments on the ISS and laboratory simulations (Ellena et al., 2024; Poughon et al., 2020).

Furthermore, space biology beyond LEO is inherently different and requires a shift in technology to enable the exploitation of organisms in autonomous experiments (Everroad et al., 2024). In this endeavor CubeSats—small, relatively inexpensive satellites—are envisioned for querying the biological effects of deep space (Harandi et al., 2022; Kanapskyte et al., 2021). In such a scenario, the development of the NASA GraviSat has led the foundation in growing microalgae and cyanobacteria on-board CubeSats during long-duration space missions (Fleming et al., 2014).

In this study, a biosensor tailored for space applications was developed leveraging a desiccation-tolerant cyanobacterium, as part of the EU-ALCYONE project aimed to realize a lab-on-chip platform for small satellites based on dried microorganisms expressing a luciferase reporter upon rehydration and space exposure (Davis et al., 2025). The desert cyanobacterium *Chroococcidiopsis* sp. CCMEE 029 has been selected due to its suitability for genetic manipulation and maintenance of exogenous plasmids after several months of air-dried storage (Billi, 2012), along with its genome stability after 1.5 years of exposure in the dried state outside the ISS (Napoli et al., 2022).

A crucial step in developing a space-tailored biosensor has been the identification of a suitable promoter to drive the reporter gene expression in *Chroococcidiopsis* sp. CCMEE 029. Several DNA-damage biosensors based on *Escherichia coli* SOS-responsive promoters have been developed (Bazhenov et al., 2023; O’Connor et al., 2025). In heterotrophic bacteria, the SOS response to DNA damage is frequently coordinated by two regulatory elements, the *recA* and *lexA* gene products. RecA binds single-stranded DNA produced upon genetic damage, promoting the self-cleavage of the LexA repressor which in turn dissociates from the SOS-box sequences leading to the expression of SOS-controlled genes (Chatterjee and Matheshwaran, 2026). On the contrary, in *Synechocystis* sp. PCC6803 LexA does not undergo auto-cleavage and is involved in various cellular processes through post-translational modification (Li et al., 2010; Takashima et al., 2020), while in *Anabaena* sp. PCC7120 LexA exhibits a RecA-independent and pH-dependent auto-cleavage and acts as a transcriptional regulator of a broad regulon of over 40 genes, including DNA repair (Kumar et al., 2018).

Hence, in this study, an *in-silico* analysis of the *Chroococcidiopsis* sp. CCMEE 029’s genome was performed with the aim to characterize its LexA homolog and evaluate its potential involvement in DNA repair processes. The presence of a putative LexA-binding motif with high similarity with the LexA-box consensus of *Anabaena* sp. PCC 7120 (Mazón et al., 2004) was searched in eight additional *Chroococcidiopsis* spp. genomes, and the binding to DNA was verified through molecular modeling and molecular dynamics simulations. The mRNA levels of the *lexA* and *recA* genes were then assessed following hydrogen peroxide (H_2_O_2_) treatment to induce DNA damage (Schipler and Iliakis, 2013). *Chroococcidiopsis* sp. CCMEE 029 was then transformed with a plasmid carrying a transcriptional fusion between a luciferase gene reporter and the upstream region of its *recA* gene containing an upstream LexA-binding motif. Transformants were tested for luminescence after H_2_O_2_-treatment, ultra-violet C (UVC) or γ-ray radiation and tested for a correlation between increasing UVC-doses and the onset of detectable DNA damage. Finally, to support future integration in the air-dried state into CubeSats and in-orbit rehydration, the biosensor was validated under simulated microgravity (SMG) conditions combined with γ-rays and its responsiveness to UVC and γ-ray irradiation verified after 4.5 months of air-dried storage.

## Materials and methods

### Strains and growth conditions

*Chroococcidiopsis* sp. CCMEE 029 (hereafter CCMEE 029) was isolated from cryptoendolithic growth in the Negev Desert (Israel) by Roseli Ocampo-Friedmann and is currently maintained at the Department of Biology, University of Rome “Tor Vergata,” as part of the Culture Collection of Microorganisms from Extreme Environments (CCMEE) established by E. Imre and Roseli Ocampo-Friedmann. For the purposes of the study, CCMEE 029 was maintained in Blue Green-11 Medium (BG-11) without shaking, at 25 °C under a constant photon flux density of 20 μmol photons m^2^ s^−1^, obtained by using a fluorescent warm-white tubular LEDs (4,000 K). Transformants were selected and grown with BG-11 containing 100 μg/mL ampicillin. *Escherichia coli* DH5α was grown in Luria–Bertani (LB) medium at 37 °C under orbital shaking, with growth media supplemented with ampicillin (100 μg/mL) when required.

### Identification of LexA in CCMEE 029 and phylogenetic analysis

LexA homologs in CCMEE 029 were searched by the TBLASTN web server using *Escherichia coli* str. K-12 substr. MG1655 LexA protein (NP_418467.1) as query and CCMEE 029 chromosome (NZ_CP083761.1) as subject. In this analysis, word size was set to 3 to improve the likelihood of finding homologs. Phylogenetic analysis was performed by aligning cyanobacterial and the *E. coli* K-12 substr. MG1655 LexA proteins, by using LexA sequences previously reported (Li et al., 2010). Alignments were performed using ClustalW in MEGA with default parameters. A phylogenetic tree was constructed using the neighbor-joining method with Poisson correction and 1,000 bootstrap replicates, with *E. coli* K-12 substr. MG1655 serving as the outgroup.

The multiple sequence alignment of LexA sequences was visualized in Jalview (v2.11.5.0) and conserved domains were annotated according to the canonical LexA domains were annotated with InterProScan (v5.74–105.0) and with NCBI’s CD-Search (Marchler-Bauer and Bryant, 2004).

### Search for LexA regulated homologs and definition of LexA-binding motif in CCMEE 029

The PCC 7120 LexA (*alr4908*), RecA (*all3272*), UvrA (*alr3716*), Ssb (*alr0088*), Alr4905, and All4790 proteins, for which a LexA-dependent regulation has been previously demonstrated (Mazón et al., 2004), were employed to find homologs in selected *Chroococcisiopsis* sp. genomes by using TBLASTN. This analysis included *Chroococcisiopsis* sp. CCMEE 029 (NZ_CP083761.1), *Chroococcidiopsis* sp. CCALA 051 (GCF_003015105.1), *Chroococcidiopsis* sp. CCNUC1 (NZ_CP097480.1), *Chroococcidiopsis cubana* CCALA 043 (GCF_003003835.1), *Chroococcidiopsis cubana* SAG 39.79 (GCF_003991895.1), *Chroococcidiopsis* sp. [FACHB-1243] (GCF_014696895.1), *Chroococcidiopsis thermalis* PCC 7203 (NC_019695.1), *Chroococcidiopsis* sp. SAG 2025 (GCF_032860985.1), and *Chroococcidiopsis* sp. TS-821 (GCF_002939305.1). Intergenic regions upstream genes coding for the detected homolog proteins were extracted, pooled, and analyzed with the Multiple Expectation maximizations for Motif Elicitation—MEME (Bailey et al., 2015) software to identify 8–20 nucleotide long palindromic motifs.

### Molecular modeling and molecular dynamics simulations

Structural models of LexA-DNA complexes were generated using AlphaFold 3 (Abramson et al., 2024). System preparation was conducted with the tleap module of AmberTools, parameterizing the systems with the ff19SB (Tian et al., 2020) and OL21 (Zgarbová et al., 2021) force fields for the protein and DNA, respectively, along with the OPC water model. Each complex was solvated in a rectangular box of OPC water molecules, maintaining a minimum buffer distance of 14 Å between the solute and the box boundaries. The systems were then neutralized and adjusted to a physiological salt concentration of 0.15 M NaCl. Under periodic boundary conditions, energy minimization was performed using the AMBER sander engine (Case et al., 2005). The process consisted of 10,000 steps (2,500 steepest descent followed by 7,500 conjugate gradient) with positional restraints applied to the solute atoms. Short-range non-bonded interactions were truncated at an 8.0 Å cutoff, while long-range electrostatics were treated using the particle mesh Ewald method (Darden et al., 1993). Following minimization, the systems were gradually heated to 313 K in the NVT ensemble using a Langevin thermostat (Goga et al., 2012). Upon reaching the target temperature, a multi-step equilibration phase was initiated, during which the harmonic restraints on the solute heavy atoms were progressively released until completely removed. Finally, system density was equilibrated in the NPT ensemble at 1.0 atm using a Monte Carlo barostat (Åqvist et al., 2004). The SHAKE algorithm (Ryckaert et al., 1977) was applied to constrain all bonds involving hydrogen atoms, permitting an integration time step of 2.0 fs. Molecular dynamics simulations were performed for a total of 200 ns in the NPT ensemble (313 K, 1.0 atm) using pmemd.cuda engine in AMBER (Salomon-Ferrer et al., 2013). Temperature and pressure were maintained throughout the production run using the Langevin thermostat and Monte Carlo barostat, respectively. Visual inspection of the molecular dynamics trajectories and molecular graphics rendering was performed using Visual Molecular Dynamics (Humphrey et al., 1996). The binding free energies of LexA-DNA complexes were estimated utilizing the MMPBSA.py module within AMBER (Miller et al., 2012). This analysis employed the non-linear Poisson–Boltzmann solver and was performed on 150 frames extracted at regular intervals from the final 150 ns of the production trajectories. For the MM-PBSA calculations, the internal and external dielectric constants were set to 8.0 and 80.0, respectively, with the ionic strength maintained at 150 mM. Buried surface area analysis was performed using the surf command in CPPTRAJ.

### Isolation of total RNA and gene expression analysis

Total RNA was extracted from three biological replicates of strain CCMEE 029 immediately after the treatment with 10 mM H_2_O_2_ for 15 and 30 min. Treated cells were harvested and centrifuged at 5,000 g for 5 min at 4 °C, the supernatant was discarded, and pellets were added with 50 μL of TRI Reagent (Sigma-Aldrich, St. Louis, MO, United States) and an equal volume of glass beads. Cells were disrupted through a Bead-Beater with three cycles for 2 min at 3500 OPM followed by 1 min on ice. After the addition of 950 μL of TRI Reagent, a last cycle of rupture through the Bead-Beater was performed. The samples were then vortexed for 15 s and left for 5 min at room temperature. A volume of 200 μL chloroform was added and the samples were mixed vigorously for 15 s and then kept for 10 min at room temperature. The suspensions were then centrifuged at 12,000 g for 15 min at 4 °C. The aqueous phase containing the total RNA was transferred to a new test tube containing 500 μL of absolute isopropanol and then mixed and left for 10 min at RT. The mixtures were centrifuged at 12,000 g for 10 min at 4 °C to precipitate the RNA as a pellet, which was then washed once with 1 mL of 75% ethanol. Following vortexing and centrifugation at 12,000 g for 15 min at 4 °C, the samples were left to dry for 5–10 min. Finally, the RNA was resuspended in 30 μL of water RNase-free (Sigma-Aldrich, St. Louis, MO, United States), reverse-transcribed into cDNA and used as template for RT-qPCR. Reactions with three technical replicates for each biological replicate were performed in 12 μL volumes, containing 10 ng of cDNA, 6 μL of iTaq™ Universal SYBR® Green Supermix (Bio-Rad Laboratories, Hercules, CA, United States), and 0.5 μM of the following primers designed to target *recA* and *lexA* genes: recAFw (5′-TTTTACGCTTCGGTACGGCT-3′) and recARv (5′-ACGGTGGGGCGACTTTATTT-3′) and lexAFw (5′-ATCGCTGCTGGTGGTTTAGT-3′) and lexARv (5′-TCGATCATGCTGTCACCCAC-3′). The 16S rRNA gene was used for normalization and amplified by using primers 16SFw (5′-TACTACAATGCTACGGACAA-3′) and 16SRv (5′-CCTGCAATCTGAACTGAG-3′) designed on the 16S rRNA gene of CCMEE 029 (GenBank accession numbers: AF279107). PCR cycling conditions were performed using a StepOnePlusTM. RealTime PCR System (Thermo Fisher Scientific, Waltham, MA, United States) as described: one step at 95 °C for 10 min, followed by 40 cycles of 95 °C for 15 s, and 60 °C for 1 min, and a ramp from 60 to 95 °C for the melting curve stage. Relative mRNA levels were calculated with the comparative cycle threshold (Ct) method and the 16S rRNA gene was used for normalization. Fold-change values were calculated by setting the values obtained from unexposed cells as 1 and considering the values from treated cells as upregulated (>1) or downregulated (<1). For each gene target reactions were conducted in triplicate.

### Plasmid construction

A shuttle plasmid has been generated by GenScript Biotech (Rijswijk, Netherlands) by using the plasmid pUC57 backbone carrying the pMB1 origin of replication and ampicillin resistance and cloning a 1,747-bp of the replication region of pDU1 plasmid from *Nostoc* sp. PCC 7524 (GenBank M80600; Walton et al., 1992), that was previously reported to be sufficient for plasmid replication in CCMEE 029 (Billi, 2012). A mutated luciferase from *Photinus pyralis* BgLuc (Calabretta et al., 2022) was cloned downstream of a 228-bp fragment upstream of the *recA* gene of CCMEE 029. The synthetic plasmid was 6,148-bp long, and was named PrecA-Luciferase that was maintained in *E. coli* DH5α in 15% glycerol stock at −80 °C.

### Cyanobacterial transformation

Strain CCMEE 029 was transformed via electroporation as previously described (Billi et al., 2001). Aliquots of 1 mL (1 × 10^8^ cells/ml) were washed once with phosphate buffered saline (PBS), twice with cold 1 mM HEPES buffer, pH 7.4, and resuspended in 50 μL of HEPES and 5 μL of PrecA-Luciferase plasmid (100 ng/μl). After electroporation 950 μL of fresh BG-11 medium was added and the transformants were incubated under optimal growth conditions for 24 h. Then 5-μl aliquots were spotted on S-Pak® Millipore mixed cellulose esters membrane filters placed on agarized BG-11 medium supplemented with 100 μg/mL ampicillin. Filters were moved on fresh selective agarized BG-11 medium every 7 days until cells were collected and analyzed as reported below.

### Transformants treatment with DNA-damaging agents

Transformants of CCMEE 029 carrying the P*recA*-Luciferase plasmid were collected from filters with a sterile loop, resuspended in 1 mL of selective BG-11 medium and exposed as three biological replicates at a cell concentration of about (1 × 10^7^ cells/mL) to the following DNA-damaging conditions: (i) UVC irradiation, aliquots of 100-μl transformants were transferred into uncovered 96-well black polystyrene microplates (Costar®, Corning) positioned at 15 cm from a UV-C lamp (Vilber Lourmat, France) providing an irradiance of 7.1 μW/cm^2^ and dose of 7.1 J/m^2^/s; (ii) γ-ray irradiation, aliquots of 1-ml transformants were transferred in Eppendorf tubes and positioned in a pool-type irradiation plant equipped with the ^60^Co gamma source (dose rate of 715 Gy/h) in high-volume shielded cell (Calliope facility, ENEA-Casaccia Research Center, Italy), after irradiation 100-μl aliquots were transferred into 96-well black polystyrene microplates; (iii) H_2_O_2_-treatment, aliquots of 1-ml transformants were resuspended in 10 mM H_2_O_2_ and after 15–30 min incubation under RT, washed with PBS and resuspended in 1 mL selective BG-11 medium, then 100-μl aliquots were transferred into 96-well black polystyrene microplates (Costar®, Corning). After the treatment the luminescence assays were performed on three technical replicates for each biological replicate as reported below.

### DNA damage evaluation

Genomic DNA was extracted and used as template for PCR as reported (Mosca et al., 2019). About 4-kbp fragment was amplified in 12-μl PCR reaction mixtures using 60 ng of DNA, 0.5 μM each of primers Chroo-4 K-2-F (5′-GCTACTCGTTGCTTTGCGTC-3′) and Chroo-4 K-2-R (5′-TTCCCCATACTTTGCTTCCCA-3′) and 6 μL of High-Fidelity Master Mix (Thermo Fisher Scientific, Waltham, MA, United States). PCR conditions were as follows: 98 °C for 3 min; 30 cycles of 98 °C for 30 s, 65 °C for 1 min, and 72 °C for 2 min; and 7 min at 72 °C. Each one of the 12-μl PCR reaction mixtures was loaded onto 1.5% agarose gel containing FluoroVue™ Nucleic Acid Gel Stain (SMOBIO Technology, Inc.), subjected to electrophoresis for about 1 h at 90 V, and visualized with a trans-illuminator.

### Simulated microgravity under γ-radiation

Transformants of CCMEE 029 in selective BG-11 medium were transported in 15 mL Falcon tubes (Corning Inc., United States) to ENEA Casaccia Research Centre (Rome, Italy) and upon arrival transferred into disposable 10-ml HARV vessels (type D-405) and the volume adjusted to 10 mL with selective BG-11 medium (cell densities about 1 × 10^6^ cells/ml). For incubation under SMG and simulated normal gravity (SNG) two Synthecon rotary cell culture systems (RCCS-2D, Cellon SA, Bereldange, Luxembourg) were positioned inside the facility each one with two vessels (two biological replicates). One apparatus rotated about a horizontal axis and the second one rotated about a vertical axis, both at 30 rpm which generates approximately 2 × 10^−2^ *g* and exposed to γ-rays for 42 min (dose rate 715 Gy/h). Controls were incubated under SMG and NG conditions without γ-ray irradiation. Following the irradiation, aliquots (three for each vessel) were pelleted and resuspended in selective BG11 medium at a cell density of about 1 × 10^7^ cells/ml. Luminescence assays were performed on the three technical replicates for each biological replicate as reported below.

### Transformants desiccation and rehydration

Transformants of CCMEE 029 were collected from filters with a sterile loop, resuspended in 1 mL of selective BG-11 medium and spotted on to hydrophilic polycarbonate filter (Isopore-Membranfilter, Isopore™). After overnight air-drying under a laminar flux, the filter was transferred into sterile 6 cm Petri dishes (Corning®, NY, United States) and stored under RT in the dark. After 4.5 months, transformants were resuspended in selective BG-11 medium and incubated for 72 h to allow recovery under optimal growth conditions.

### Luciferase assay

Luciferase activity was quantified in 96-well black polystyrene microplates by adding 10 μL of 5 mM D-Luciferin substrate (D-Luciferin sodium salt, Sigma-Aldrich, 2024 Merck KGaA, Darmstadt, Germany) to 100-μl aliquots of CCMEE 029 transformants, which after each treatment were normalized by optical density readings at 730 nm, at a cell density of about 1 × 10^7^ cells/ml. Luminescence measurements were obtained in triplicate for each biological replicate using a TECAN Infinite M Plex microplate reader (Tecan Group Ltd., Männedorf, Switzerland) or a Varioskan LUX Multimode Microplate Reader (Thermo Fisher Scientific Inc.), the latter available at ENEA Casaccia Research Centre (Rome, Italy). Background signal (blank) was subtracted from all readings and data were expressed in relative light units (RLU).

### Statistical analysis

Each PCR experiment included three biological replicates, each one with three technical replicates and shown as means with standard deviation (SD) and significance was assessed by using Student’s *t*-test and expressed as mean ± SD. For luminescence assays with three technical replicates for each one of the three biological replicates were averaged. For the microgravity experiment two biological replicates were used, and three technical replicates were averaged. A two-way ANOVA was performed considering treatment and time as independent factors, while luminescence was used as the dependent variable. Variability among technical replicates was first calculated as SD, and the standard error of the mean (SEM) was subsequently derived for graphical representation. Error bars represent the mean SD of technical replicates. Statistical significance was assessed based on *p*-values obtained from the two-way ANOVA and reported in the figures using asterisks (^****^p < 0.001, ^*^p < 0.05, ^ns^*p* > 0.05).

## Results

### *In-silico* identification of LexA in CCMEE 029 and phylogenetic analysis

**Figure 1:**
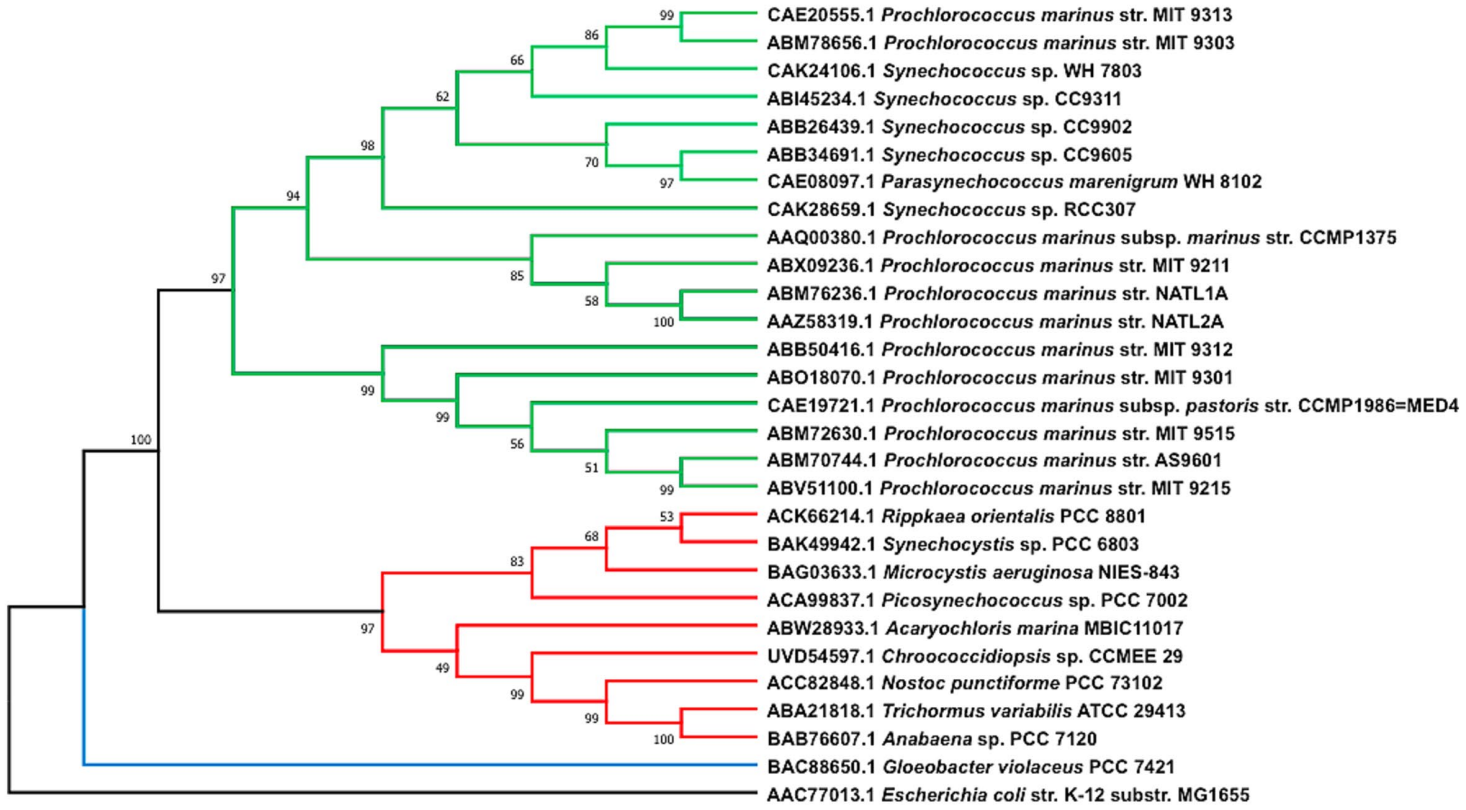
Phylogenetic relationships of selected cyanobacterial LexA proteins. The tree is rooted to the LexA of *E. coli* K12 strain MG1655 and represents the consensus obtained after 1,000 replicates. Bootstrap values are reported next to the branches in percentage. Clade A, B, and C are colored in blue, green, and red, respectively. Codes indicate NCBI GenBank accession numbers.

Analysis of *Chroococcidiopsis* sp. CCMEE 029’s genome identified the presence of a LexA homolog (ChLexA) exhibiting 80.7, 48.5, and 33.7% overall amino acid sequence identity with LexA from *Anabaena* sp. PCC 7120 (AnLexA), *Synechocystis* sp. PCC 6803 (SyLexA), and *E. coli* (EcLexA), respectively. A phylogenetic tree, including previously reported LexA proteins (Li et al., 2010) and the CCMEE 29 homolog, revealed that the ChLexA clustered in a sub-group of Clade B, which included AnLexA (Figure 1).

**Figure 2:**
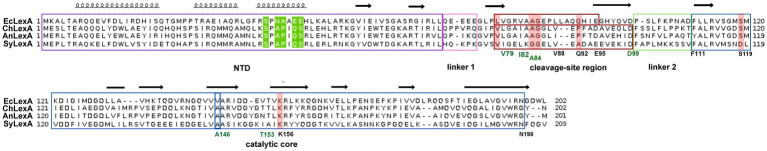
Amino acid sequence alignment and secondary structure prediction of *Chroococcidiopsis* sp. CCMEE 029’s LexA. N-terminal region with three α-helices (indicated by spirals) with DNA-contacting residues in helix III (shaded in green) and 2 β-strands (indicated by arrows). C-terminal region with nine β-strands (indicated by arrows).

The multiple alignment analysis of ChLexA with AnLexA, SyLexA, and EcLexA revealed the presence of shared key features (Figure 2). The N-terminal domain (NTD, residues 1–69, *E. coli* numbering) exhibits three alfa-helices with the second and third forming a helix-turn-helix motif reported to contain the repressor DNA-binding domain (Zhang et al., 2010). In EcLexA, residues Ser39, Asn41, Ala42, Glu44, and Glu45 are involved in DNA binding (Knegtel et al., 1995). In contrast, ChLexA, as well as AnLexA and SyLexA, retain Ser39 but exhibit substitutions at the corresponding positions, namely Ala41, Pro42, Gln44, and Ser45, consistent with what has been reported for other cyanobacterial LexA proteins (Li et al., 2010).

The NTD of ChLexA is connected by a short flexible linker (residues 70–74, *E. coli* numbering) to the C-terminal domain (CTD; residues 75–202 *E. coli* numbering), that in *E. coli* contains the cleavage site Ala84-Gly85 within the cleavage-site region (CSR) spanning from L78 to E95 (Luo et al., 2001). In ChLexA and AnLexA, the Ala84-Gly85 cleavage site is present while it is absent in SyLexA where the Ala84 residue is replaced by Gly84 resulting in a non-cleavable site (Oliveira and Lindblad, 2011). Compared to EcLexA, ChLexA has a longer CSR spanning from V78 to D99 containing the 86-GLV-88 residues in agreement with the conserved 86-GLI-88 residues reported as essential for the cleavage of AnLexA and 86-GLI/V-88 residues of cleavable cyanobacterial LexA, that is absent in SyLexA (Kumar et al., 2015).

ChLexA and AnLexA present the catalytic core region (from Phe111 to Asn198) reported to be responsible in EcLexA for the nucleophilic attack required for LexA cleavage between Ala84 and Gly85 (Luo et al., 2001) and including the Ser119-Lys156 (S119- K156), that is absent in SyLexA. Each cyanobacterial LexA lacks the Q92 residue essential for RecA-dependent cleavage in EcLexA. ChLexA showed the substitutions of four residues (L79, I82, A146, T153) out of the six residues identified in EcLexA as involved in RecA binding and autocleavage, namely V79, V82, F111, L113, V146, and V153 (Cory et al., 2024), which are substituted also in AnLexA and SyLexA (Figure 2).

### *In-silico* prediction of LexA-binding motifs in CCMEE 029

**Figure 3:**
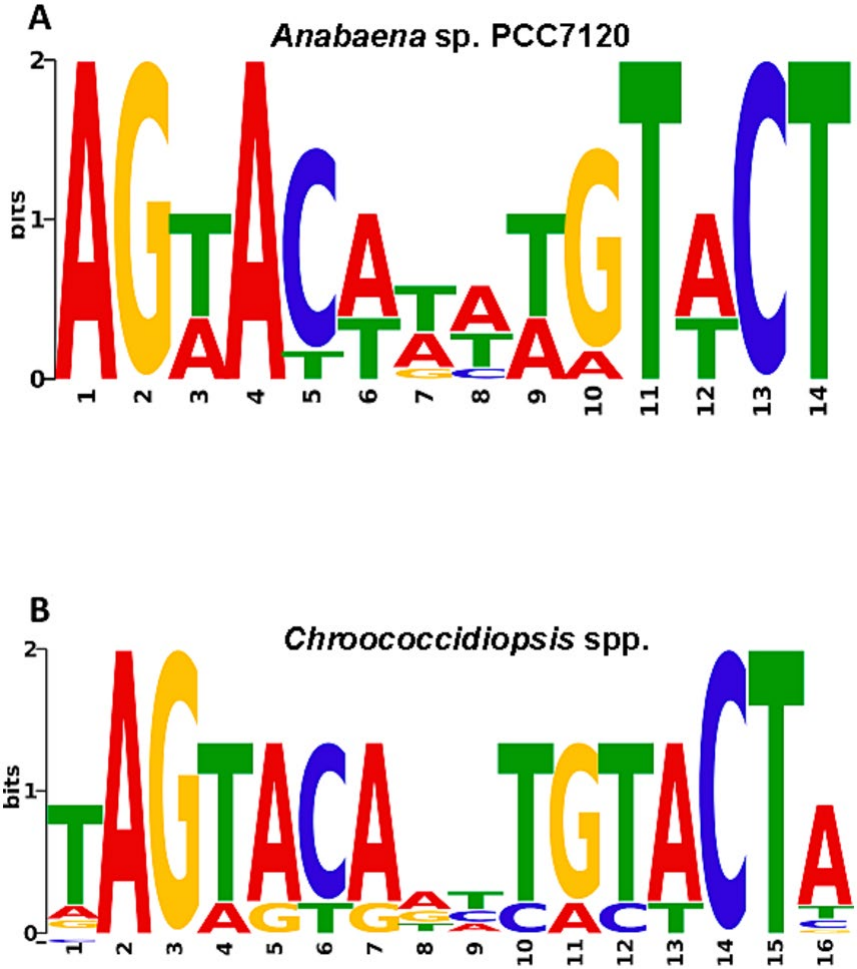
Motifs identified by a MEME analysis of the *lexA* (*alr4908*), *recA* (*all3272*), *ssb* (*alr0088*), and *uvrA* (alr3716) upstream regions of *Anabaena* sp. PCC7120 **(A)** and of the upstream regions of their homologs in nine *Chroococcidiopsis* spp. genomes **(B)**.

The search for homologs of six *Anabaena* sp. PCC 7120 proteins for which a LexA-dependent regulation has been demonstrated (Mazon et al., 2004), revealed the presence of LexA-, RecA-, UvrA-, and Ssb-like proteins in all the nine analyzed *Chroococcisiopsis* spp. genomes. A MEME analysis of the upstream regions of these set of genes identified a conserved motif located upstream of the *lexA*, *recA*, and *ssb* genes of all the *Chroococcisiopsis* spp. genomes, which was very similar to that obtained analyzing the corresponding set of sequences of *Anabaena* sp. PCC 7120 (Figures 3A,B). In detail, the motif found for *Anabaena* sp. PCC 7120 (AGWA-N6-TWCT) overlapped to the previously reported conserved consensus AGT-N_4-11_-ACT (Mazón et al., 2004), while that of CCMEE 029 was AGWRYR-N_2_-YRYWCT. Notably, in both motifs, identical left (AGW) and right (WCT) arms are separated by an 8-nucleotide spacer. Building on these results, putative LexA-regulated genes in CCMEE 029 were searched by screening the upstream regions of 22 genes encoding homologs for which LexA binding in *Anabaena* sp. PCC 7120 was experimentally validated (Srivastava et al., 2022; Kumar et al., 2018; Srivastava et al., 2023; Mazón et al., 2004). Putative LexA-boxes were identified by using the AGT-N_4-11_-ACT motif (Kumar et al., 2018) or when matches to a less stringent previously described motif (Srivastava et al., 2022) were found. This analysis revealed the presence of putative LexA binding sites in 22 of the 33 homologs present in CCMEE 029 (Supplementary Table S1).

### Molecular modeling and dynamics simulations LexA-binding

**Figure 4:**
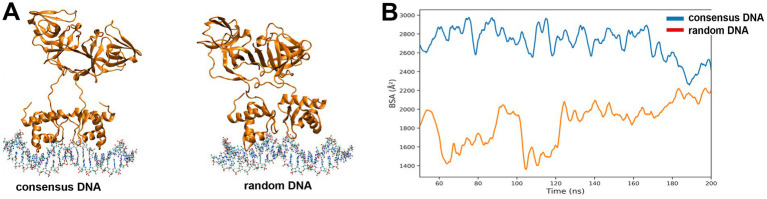
LexA-DNA binding in *Chroococcidiopsis* sp. CCMEE 029. Representative structural snapshots of the complexes at the 120 ns time point of the binding between the LexA homodimer and consensus DNA motif (left) or random DNA sequence (right) **(A)**. Buried surface area calculation for the LexA-consensus DNA and the LexA-random DNA complexes evaluated over the last 150 ns of the simulation time **(B)**.

To investigate the interaction dynamics, structural models of two distinct LexA-DNA systems were generated. The first model comprised a 22-base pair double-stranded DNA fragment containing the LexA DNA consensus sequence (as occurring upstream of the *recA* gene of CCMEE 029) flanked by a 4-base pair region on each side (5′-aaatAGTATATCTGCACTagtc-3′). In both setups, LexA was modeled in its functional homodimeric state bound to the DNA target. The final all-atom setups for the consensus and control systems contained 141,225 and 136,816 atoms, respectively, with an initial net charge of −42 before neutralization. The trajectories obtained over 200 ns suggest that LexA maintains a more stable interaction with the consensus sequence than with the random DNA sequence (Figure 4A). The quantification of the thermodynamic affinity over the last 150 ns of the simulation time revealed a more favorable binding free energy for the consensus sequence (ΔGbind = −92.76 ± 2.38 kcal/mol) than with the random DNA sequence (ΔGbind = −65.73 ± 2.10 kcal/mol).

To further characterize the extent of the protein–DNA interface, the buried surface area was evaluated over the same time interval. The LexA-consensus DNA complex exhibited consistently higher values than the random sequence, indicating a broader and tighter interaction interface, consistent with the favorable binding free energies (Figure 4B).

### Expression of the *lexA* and *recA* genes in response to DNA-damaging agents

**Figure 5:**
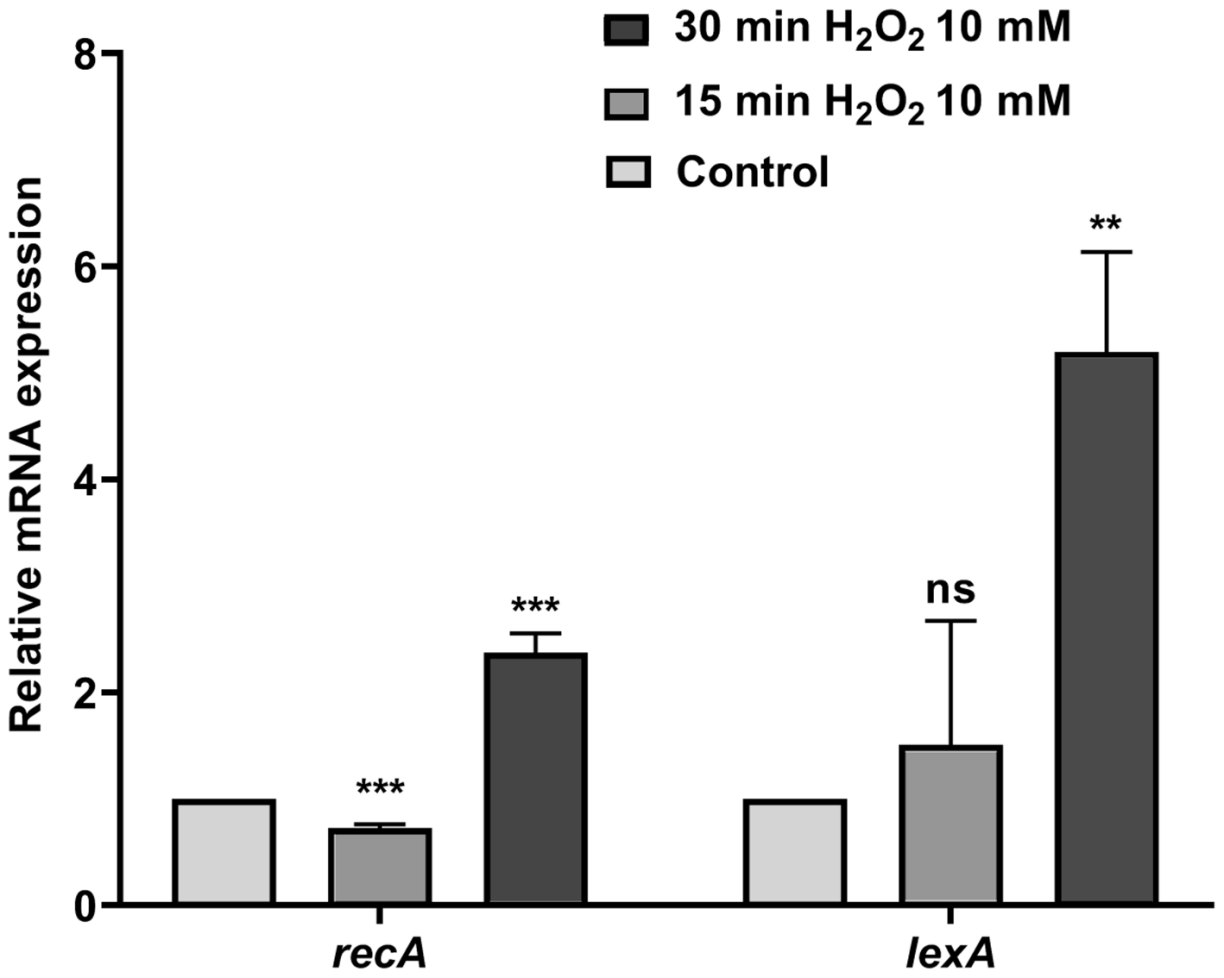
Expression of the *lexA* and *recA* genes in *Chroococcidiopsis* sp. CCMEE 029. Cells were treated with 10 mM H_2_O_2_ for 15 and 30 min. Control: unexposed cells. *nsp* > 0.05, **p < 0.01, ***p < 0.001.

The transcriptional changes of the *lexA* and *recA* genes of *Chroococcidiopsis* sp. CCMEE 029 were investigated after exposure to 10 mM H_2_O_2_ for 15 min and 30 min. The transcript levels of both *lexA* and *recA* genes significantly increased after the 30-min treatment, while no significant difference was observed after treatment with 10 mM H_2_O_2_ for 15 min (Figure 5).

### Biosensor construction and responsiveness to DNA-damaging agents

**Figure 6:**
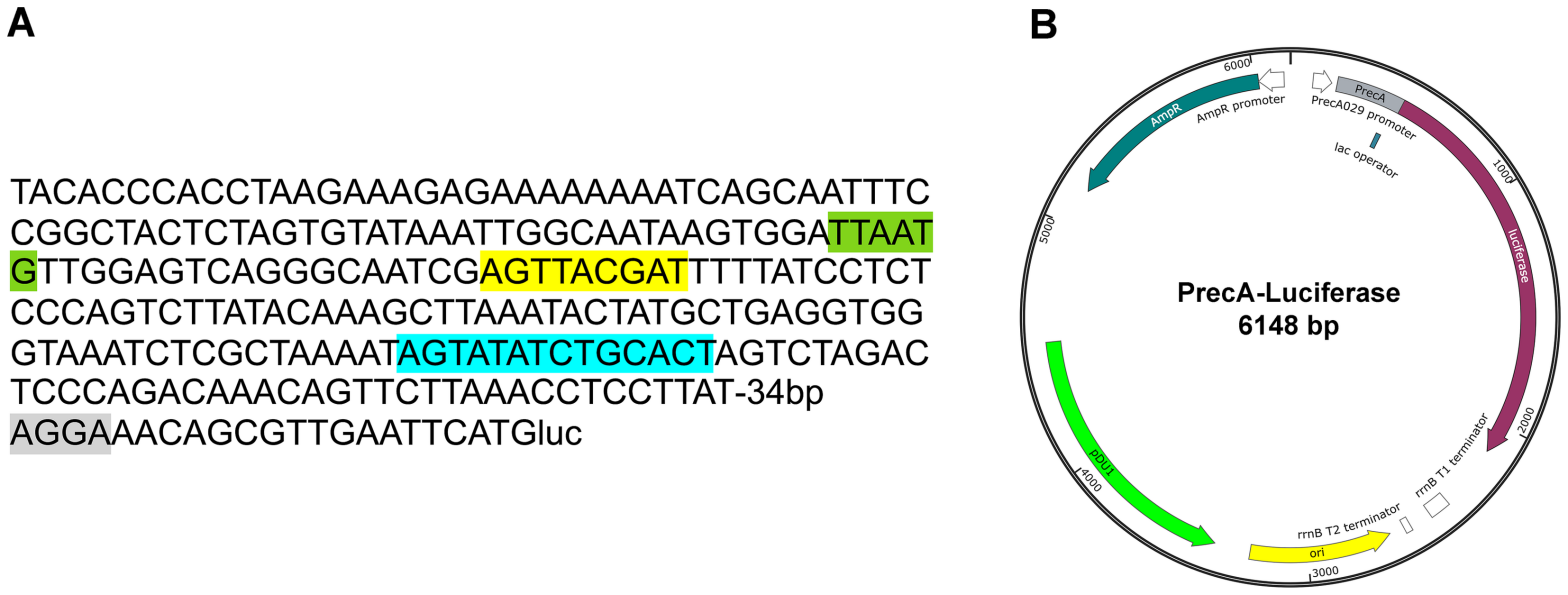
PrecA-Luciferase shuttle plasmid. Upstream region (228 nt) of the *recA* gene of *Chroococcidiopsis* sp. CCMEE 029 driving the BgLuc expression, putative - 10 box (AGTTACGAT shaded yellow), - 35 box (TTAATG shaded green), LexA-binding motif (AGTATATCTGCACT shaded blue) and RBS (AGGA, shaded gray) **(A)**. Map of the shuttle plasmid derived from pUC57 backbone carrying pMB1 and pDU1 sequences for replication in *E. coli* and *Chroococcidiopsis* sp. CCMEE 029 **(B)**.

Following the identification of the *recA* gene of CCMEE 029 the 228-bp region with the LexA-binding motif upstream (Figure 6A) was used to realize a transcriptional fusion with BgLuc encoding a mutated luciferase from *Photinus pyralis* (Calabretta et al., 2022). The PrecA-Luciferase plasmid carrying the transcriptional fusion was a shuttle plasmid capable of replication in *E. coli* and contained a 1.75 kbp-pDU1 fragment for replication in *Chroococcidiopsis* sp. CCMEE 029, along with ampicillin selection marker (Figure 6B).

**Figure 7:**
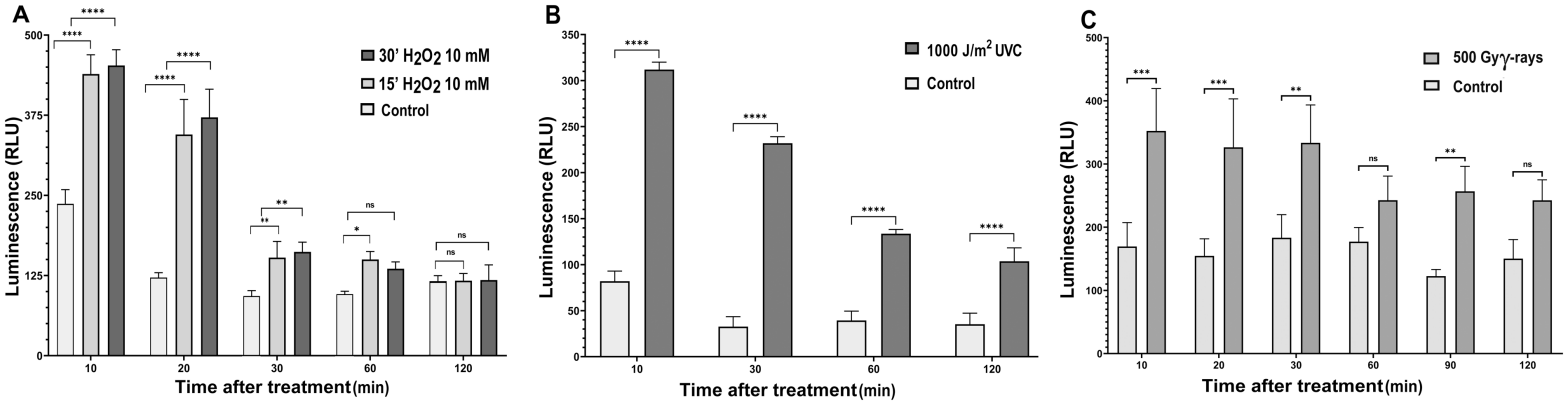
Responsiveness of *Chroococcidiopsis* biosensor (P*recA*: BgLuc) to DNA-damaging conditions. Luminescent signal after 30-min treatment with 10 mM H_2_O_2_ **(A)**. Luminescent signal after exposure to 1,000 J/m^2^ of UVC radiation **(B)**. Luminescent signal after irradiation with 500 Gy of γ-rays (dose rate 715 Gy/h) **(C)**. Control: untreated biosensor. *nsp* > 0.05, *p < 0.05, **p < 0.01, ***p < 0.001, ****p < 0.0001.

After electroporation transformants (PrecA: Luciferase) were selected on BG-11 medium containing ampicillin and tested for responsiveness to DNA-damaging agents by monitoring the luminescent signal after luciferase substrate addition (Figure 7). A luminescent signal occurred after 15- and 30-min treatment with 10 mM H_2_O_2_ that was significantly higher than unirradiated control 10 and 20 min after the luciferase substrate addition (Figure 7A). The irradiation with 1,000 J/m^2^ of UVC induced a luminescent signal that was significantly higher than unirradiated control, and that progressively decreased during the 120-min recording of the luminescence (Figure 7B). After irradiation with 500 Gy of γ-rays a luminescent signal occurred that was significantly higher than the un-irradiated control, up to 120 min after the irradiation (Figure 7C).

### Correlation between DNA damage and biosensor signal

**Figure 8:**
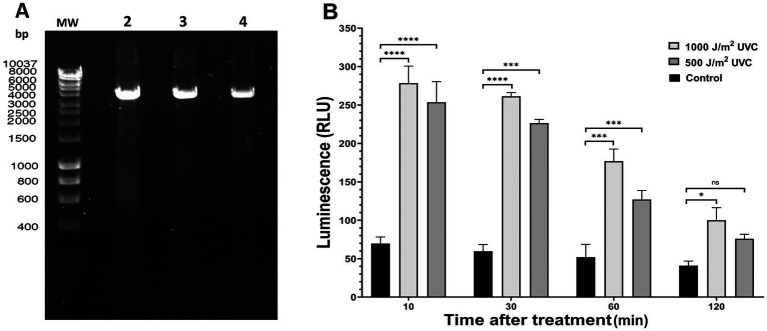
Assessment of DNA damage and responsiveness of *Chroococcidiopsis* biosensor (P*recA*: BgLuc) under UVC. PCR amplification of a 4-kbp genomic fragment in control (lane 2), in cells irradiated with 500 J/m^2^ of UVC (lane 3) and cells irradiated with 1,000 J/m^2^ of UVC (lane 4); lane 1: Hyperladder 1 kbp (Bioline Meridian Life Science, Memphis, TN, United States) **(A)**. Luminescent signal after exposure of the biosensor to 500 and 1,000 J/m^2^ of UVC radiation **(B)**. *nsp* > 0.05, *p < 0.05, ***p < 0.001, ****p < 0.0001.

The induction of DNA damage in *Chroococcidiopsis* sp. CCMEE 029 was qualitatively evaluated after irradiation with increasing UVC doses by testing the genomic suitability as template in PCR amplifications of a 4-kbp sequence target (Figure 8A). An amplicon of the expected size was obtained from control and UVC-irradiated samples, and the intensity of the PCR band from cells irradiated with 500 J/m^2^ (Figure 8A, lane 3) and cells irradiated with 1,000 J/m^2^ (Figure 8A, lane 4) showed a progressively reduced intensity compared to control (Figure 8A, lane 2). After irradiation with 500 and 1,000 J/m^2^ of UVC a significantly higher luminescent signal was emitted by the biosensor compared to non-irradiated control (Figure 8B).

### Biosensor responsiveness under simulated microgravity

**Figure 9:**
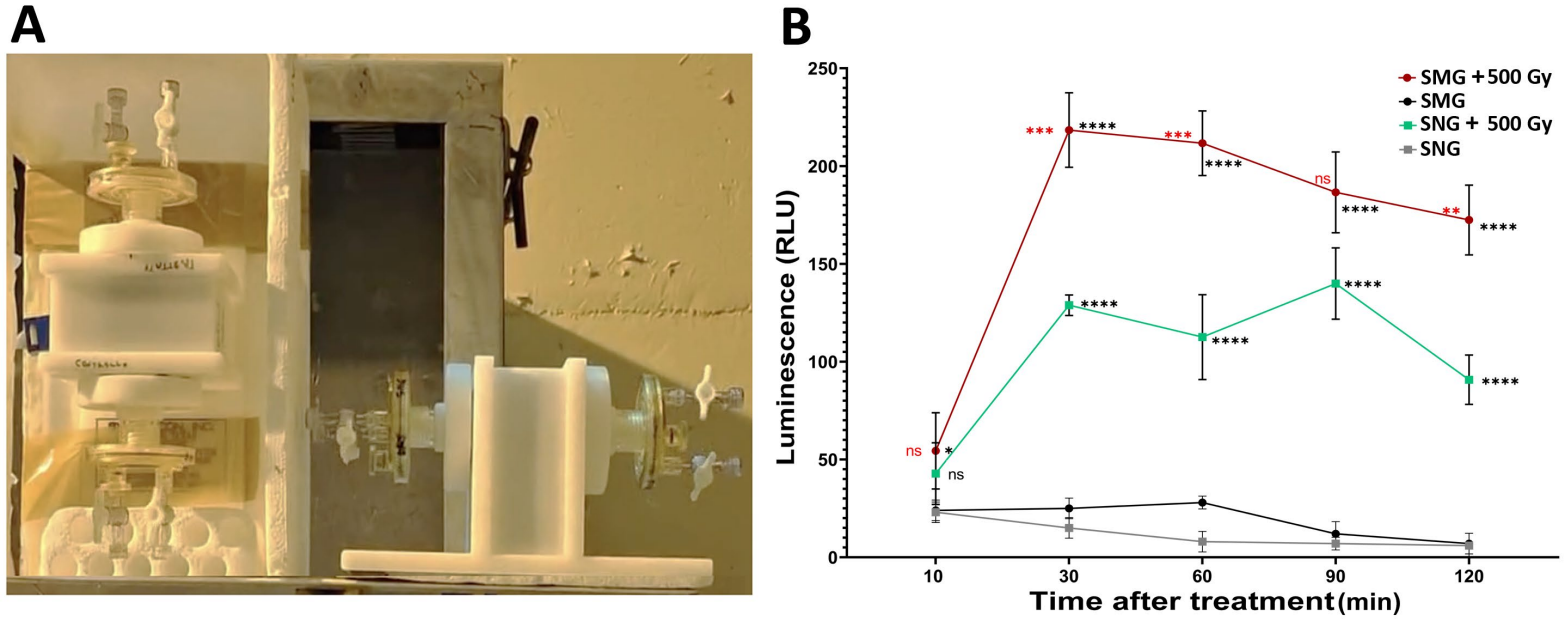
Simulated microgravity under γ-rays. Image of the two RCCS-2D positioned inside the Calliope γ-ray irradiation facility **(A)**. Luminescent signal of the biosensor under SMG and SNG combined to 500 Gy of γ-rays (dose rate 715 Gy/h) **(B)**. *nsp* > 0.05, ***p < 0.001, ****p < 0.0001.

The developed cyanobacterial biosensor (PrecA: Luciferase) was tested for responsiveness to DNA-damaging conditions by performing γ-ray irradiation under SMG and control SNG using two 2-D RCCS systems (Figure 9A). The signal emitted by the irradiated biosensor was higher than control, i.e., unirradiated biosensor under SMG and SNG. The biosensor irradiated with γ-rays under SMG emitted a luminescent signal statistically higher than the biosensor irradiated under SNG (Figure 9B).

### Responsiveness of rehydrated biosensor to DNA-damaging conditions

**Figure 10:**
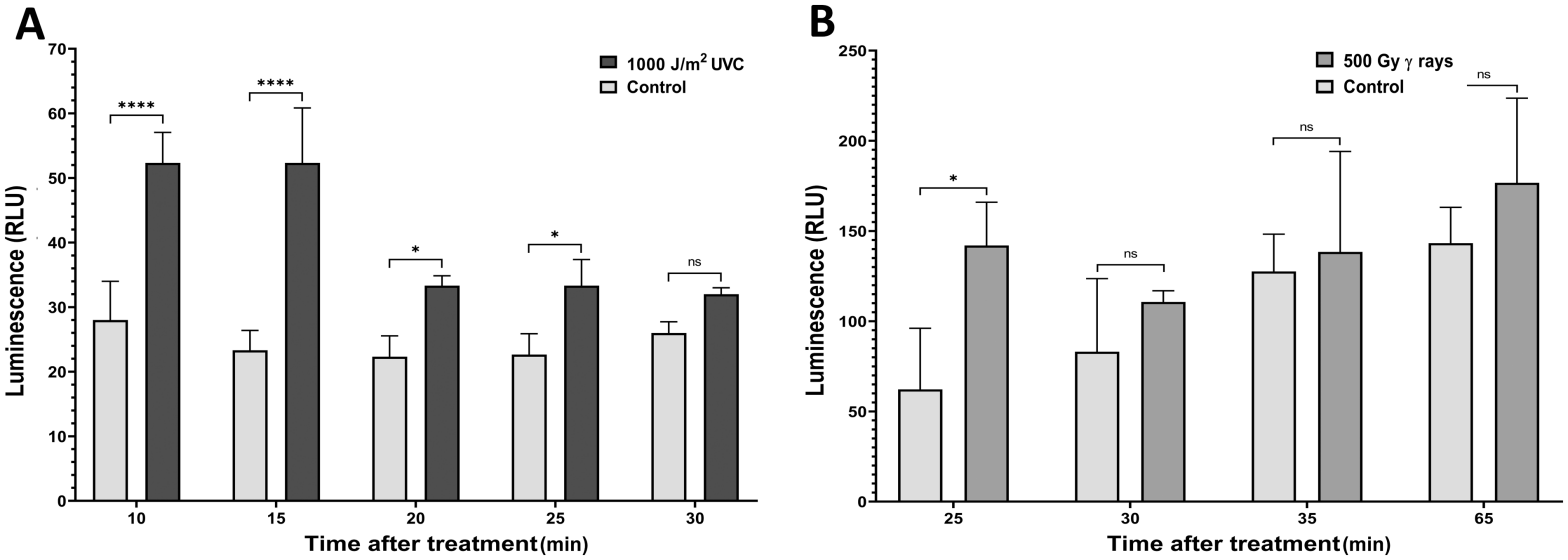
Responsiveness of the *Chroococcidiopsis* biosensor (P*recA*: BgLuc) to DNA-damaging agents after 4.5 months of air-dried storage. Rehydrated biosensor irradiated with 1,000 J/m^2^ of UVC **(A)**. Rehydrated biosensor irradiated with 500 Gy of γ rays (dose rate 715 Gy/h) **(B)**. Control: rehydrated biosensor. *nsp* > 0.05, *p < 0.05, ****p < 0.0001.

After storage in the air-dried state for 4.5 months the responsiveness of the reactivated cyanobacterial biosensor (P*recA*: BgLuc) was tested. Following 72 h of rehydration in selective BG-11 medium the rehydrated biosensor was irradiated with UVC and γ-rays (Figure 10). A luminescent signal 2-fold higher than that of rehydrated, unirradiated biosensor was detected 10 and 15 min after the irradiation with 1,000 J/m^2^ of UVC (Figure 10A). The irradiation with 50 Gy of γ-rays of the rehydrated biosensor induced the emission of a luminescent signal that was almost 3-fold higher than that of rehydrated, unirradiated biosensor 25 min after the irradiation, although over the time the control signal increased (Figure 10B).

## Discussion

In this study a LexA-mediated response biosensor tailored for space applications was developed by leveraging the desert cyanobacterium *Chroococcidiopsis* sp. CCMEE 029. The identification in its genome of the *lexA* gene, provided the first requisite toward a biosensor development using an SOS-based promoter (e.g., *recA* promoter) to drive the expression of a reported gene, since cyanobacteria lacking the *lexA* gene have been identified (Li et al., 2010). An additional requisite was provided by the phylogenetic relationships of LexA of CCMEE 029 with other previously identified cyanobacterial LexA (Li et al., 2010) along with the bioinformatic analysis of conserved domains through comparison with LexA of *Synechocystis* sp. PCC6803 and *Anabaena* sp. PCC7120. Results showed that LexA of CCMEE 029 shared key structural features with the homologs in *Anabaena* sp. PCC 7120 rather than in *Synechocystis* sp. PCC 6803. Notably, in *Synechocystis* sp. PCC6803 LexA does not undergo auto-cleavage and it is not involved in DNA repair (Takashima et al., 2020), while in *Anabaena* sp. PCC7120 it exhibits a RecA-independent and pH-dependent auto-cleavage and regulates the expression of several genes in response to multiple abiotic stress, including a few DNA repair genes (Kumar et al., 2015; Kumar et al., 2018).

Even though in this study the DNA-binding capability and autocleavage of LexA of CCMEE 029 has not been tested *in vivo*, it might be supported by the presence of the same residue substitutions as reported for other cyanobacteria, including *Anabaena* sp. PCC 7120 (Kumar et al., 2015). Moreover, the DNA binding of CCMEE 029′ LexA was confirmed by molecular modeling and molecular dynamics simulations, that showed how the binding of CCMEE 029’s LexA with the consensus motif (AGTATATCTGCACT) present upstream of the *recA* gene was consistently more stable than with a random sequence. Furthermore, the *in-silico* analysis of CCMEE 029’s genome identified putative LexA binding motifs in the 5′ flanking regions of 22/33 genes encoding for *Anabaena* sp. PCC 7120 homologs for which LexA binding has been previously demonstrated (Kumar et al., 2018).

The presence of a LexA-box upstream of the *lexA* and *recA* genes of CCMEE 029 along with their overexpression after treatment for 30 min with 10 mM H_2_O_2_, further supported the development of a LexA-mediated DNA-damage biosensor, although a more global stress management role for LexA remains to be investigated. The scored overexpression might be due to an increased cytoplasmic pH, facilitating LexA auto-cleavage as reported for *Anabaena* sp. PCC7120. A direct correlation indicating a pH increase during oxidative stress has not been shown in cyanobacteria, while the induction of the SOS response in alkaline pH has been shown for *E. coli* (Schuldiner et al., 1986). In *Anabaena* sp. PCC7120 the regulation by LexA might not follow the typical de-repression due to LexA auto-cleavage as suggested by the absence of RecA-binding sites, as well as by an *in-vitro* cleavage independent of RecA and dependent on increased cytoplasmic pH (Kumar et al., 2015).

When CCMEE 029 was engineered with a plasmid carrying a transcriptional fusion between the upstream region of its *recA* gene containing the LexA-box and a firefly luciferase, transformants emitted a luminescent signal following H_2_O_2_ treatment or irradiation with UVC and γ-rays. A correlation between increasing UVC doses and the onset of DNA damage as revealed by PCR amplification of an about 4-kbp genome fragment has been detected. The observed background signal in the absence of SOS-inducing agents, has been reported also in *E. coli* SOS-biosensors and ascribed to the activation of the P*recA* promoter due to spontaneous DNA damage, which triggers the SOS response (Abilev et al., 2020). Lower basal levels might be obtained in *Chroococcidiopsis* biosensors by using promoters with stronger LexA-binding properties. Indeed *E. coli* biosensors based on SOS promoters such as *cdA*, *umuDC*, *sulA*, and r*ecA* resulted in progressively increased basal levels due to LexA-binding properties, the *cda* promoter having two strong SOS boxes as opposed to *recA* and *sulA* promoters with one SOS box (Norman et al., 2005). Reduced basal levels might result also from a low-copy-number reporter vector that prevents a titration effect of LexA (sequestered LexA) (Norman et al., 2005; Mora-Garduño et al., 2022). However, the copy number of pDU1-based plasmids in cyanobacteria is uncertain and reported to range from one to 17 copies for *Anabaena* sp. strain PCC 7120 (Lang and Haselkorn, 1991; Lee et al., 2003).

In *Chroococcidiopsis* biosensors the background luminescent signal after H_2_O_2_-treatment and UVC radiation remained low and constant over time, whereas controls in the experiments of γ-ray irradiation increased over time. This might be attributed to the transport of transformants to the irradiation facility under non-optimal growth conditions which might have affected their metabolism thus inducing the LexA de-repression from upstream regions of genes responsive to abiotic stress rather than DNA damage. Indeed, among the 22 genes identified in *Chroococcidiopsis* sp. CCMEE 029’s genome with a LexA-binding motif, one gene was encoding a cytochrome b6-f complex subunit (orthologous to asl4754) and involved in transferring electrons from photosystem II to photosystem I. Indeed, in cyanobacteria LexA acts as a global regulator of metabolic and abiotic stress responses rather than a DNA-damage (SOS) response regulator (Kumar et al., 2018).

For space application the dependency on the exogenous addition of luciferin substrate for signal detection may complicate autonomous operation in space-based platforms. Autonomously bioluminescent cyanobacteria were developed by integrating the *luxAB* and *luxCDE* genes encoding for luciferase and its substrate into two neutral sites of their genome (Mackey et al., 2007). A self-sustained bioluminescent reporter system is not of easy development for *Chroococcidiopsis* sp. CCMEE 029 due to the absence of validated neutral sites as well as of a second origin of replication compatible with the pDU1 to allow the co-transformation with two plasmids. Nevertheless, the requirement for exogenous addition of luciferin might not be limiting the integration of the dried biosensor in CubeSats like the AstroBio-CubeSat that was previously validated for chemiluminescence-based astrobiology experiments in space (Calabria et al., 2023). Indeed, the integration requires a microfluid system with two reservoirs, one for the injection of the growth medium to reactivate the dried biosensor and one for the luciferin. A similar two-step approach has been developed for the NASA BioSentinel CubeSat in which dried cells of *Saccharomyces cerevisiae* were first rehydrated and then tested for viability upon the injection of the AlamarBlue (Lingam et al., 2025).

The biosensor responsiveness was verified under SMG conditions combined with γ-rays that were utilized as a proxy of the space environment. Notably, under ionizing radiation the biosensor incubated in SMG emitted an enhanced luminescent signal compared to SNG. The enhanced response seems in agreement with a synergic effect of microgravity and irradiation in inducing DNA damage (Moreno-Villanueva et al., 2017), thus establishing a first groundwork for a cyanobacterial biosensor for space applications. Notably, the biosensor responsiveness maintained after 4.5 months of air-dried state storage provides a requisite for future integration into CubeSats and in-orbit reactivation and luciferase-based detection of space effects.

## Data Availability

The datasets presented in this study can be found in online repositories. The names of the repository/repositories and accession number(s) can be found at: NZ_CP083761.1.
