# Supplementary material for: Harnessing desiccation-tolerant cyanobacteria as biosensors for LexA-mediated responses in deep space
Source: Front Microbiol. 2026 Jul 15;17:1842062. doi: 10.3389/fmicb.2026.1842062 (PMC13416735; doi:10.3389/fmicb.2026.1842062)
Supplement: Supplementary file 1 [file Table_1.DOCX]

**Table 1.** Putative ChLexA-boxes located upstream of genes for which LexA binding has been previously demonstrated in homologs from *Anabaena* sp. PCC7120. Left and right arms of ChLexA-boxes are in bold and palindromic regions in red.

| **PCC 7120 Gene annotation (symbol)** | **CCMEE 029 Protein_id** | **CCMEE 029 Annotation** | **% Identity (% Coverage)^a^** | **ChroLexA-box^b^** | **No of bases upstream ATG^c^** | **Sequence of -10 region^d^** | **No of bases upstream of ATG^e^** |
| --- | --- | --- | --- | --- | --- | --- | --- |
| *all0008* (*atpF2*) | WP_250125932.1 | F0F1 ATP synthase subunit B' | 100 (82) | **AGG**GGGAATGC**CCT** | 104 | CTTTACGGT | 65 |
| *alr0020* (*apcE*) | WP_250125938.1 | phycobilisome rod-core linker polypeptide | 78 (99) | TA**AGT**AATTAAAGTG**ACT**TA | 62 | ACTTATATT | 292 |
| *alr0088* (*ssb1*) | WP_250122517.1 | single-stranded DNA-binding protein | 80 (89) | TG**AGT**AGCCCTAC**ACT**TG TC**AGT**TTAAGCG**ACT**GG AG**AGT**ACACTTGT**ACT**GA | 110 64 14 | GTGTAAACT CGCTATGAT | 376 43 |
| *alr0525* (*pecC*) | WP_250125846.1 | phycobilisome linker polypeptide | 42 (99) | CT**AGC**AGTT**GCT**GA | 79 | TGTTTTTCT | 55 |
| *alr0528* (*cpcB*) | WP_250125844.1 | phycocyanin subunit beta | 83 (98) | TA**AGT**ATAAAT**ACT**AT AA**AGT**TAAACAAA**ACT**AT AA**AGT**GTTTG**ACT**TG | 387 210 88 | ATATAAACT TGATAAAAC | 419 52 |
| *alr0803* (-) | WP_250121586.1 | general stress protein | 57 (99) | CG**AGA**TACCTCTTG**TCT**CT | 143 | AGCCAAACT | 80 |
| *all0875* (*glgB*) | WP_250125343.1 | alpha-amylase family glycosyl hydrolase | 82 (100) | AC**AGA**AGTTTAGCTCT**TCT**GC | 13 | GGTTATTTT | 76 |
| *alr1524* (*rbcL*) | WP_250123779.1 | form I ribulose bisphosphate carboxylase large subunit | 92 (100) | GA**AGT**AATTT**ACT**TC | 15 | TATTAAAAT | 82 |
| *alr1742* (*dnaK*) | WP_250122389.1 | molecular chaperone DnaK | 88 (100) | AG**AGC**GATC**GCT**AT GT**AGC**TGTACAA**GCT**CA CC**TGT**ATTTTCTTGA**ACA**GT | 385 134 33 | GATTAAACT CGCTATAAA | 70 380 |
| *alr2328* (*glnA*) | WP_250121916.1 | type I glutamate--ammonia ligase | 79 (100) | GA**AGT**TATTCTCCCA**ACT**AT | 352 | TAGTAGAAT | 56 |
| *all2919* (*fd2*) | WP_250125522.1 | 2Fe-2S iron-sulfur cluster-binding protein | 78 (100) | CA**TGT**AAGAAAATAT**ACA**GT TA**AGA**TTATGAAGC**TCT**TA GG**AGG**GATTCGAAC**CCT**CG | 383 303 149 | TCATAAAAT AGTTATTTT | 335 29 |
| *all3272* (*recA*) | WP_250121493.1 | recombinase RecA | 86 (100) | AT**AGT**ATATCTGC**ACT**AG CT**AGT**CTAG**ACT**CC | 52 42 | AGTTACGAT | 133 |
| *alr3421* (*petB*) | WP_250121697.1 | cytochrome b6 | 95 (100) | TA**AGG**CGCTTC**CCT**TT TC**AGC**TACTTTCGATT**GCT**GA | 210 130 | TGTTACTTT | 172 |
| *all4121* (*petH*) | WP_250124343.1 | ferredoxin--NADP reductase | 73 (100) | TG**AGT**GATTTTTTA**ACT**TG TA**AGT**ATTTAATCCT**ACT**GT | 498 353 | CTGTAAGCT | 207 |
| *alr4123* (*prk*) | WP_250124342.1 | phosphoribulokinase | 86 (100) | AC**AGT**AGGATTAAAT**ACT**TA CA**AGT**TAAAAAATC**ACT**CA | 167 23 | TTTTATAAA | 282 |
| *asl4263* (*petN*) | WP_250126076.1 | cytochrome b6-f complex subunit PetN | 96 (97) | GG**AGT**CTTCACAA**ACT**AT | 66 | CGGTATGAT | 42 |
| *alr4404* (*ahpC*) | WP_250122264.1 | peroxiredoxin | 82 (100) | AA**AGT**AGAAGA**ACT**AA | 30 | ATGTATAAT | 199 |
| *alr4641* (*prxA*) | WP_250122746.1 | peroxiredoxin | 86 (100) | AC**AGT**TCGTAATTA**ACT**AA | 81 | GGGTAAGTT | 57 |
| *all4713* (*galE*) | WP_250126123.1 | UDP-glucose 4-epimerase GalE | 79 (100) | GT**AGT**GGCTGGA**ACT**CA TT**AGT**GGTTTTGCT**ACT**CC | 325 91 | CTGTATTAT | 266 |
| *all4790* (*recG*) | WP_250124816.1 | hypothetical protein | 55 (100) | TT**AGT**TTTA**ACT**GG | 42 | GGTTATCCT | 33 |
| *alr4908* (*lexA*) | WP_250121220.1 | transcriptional repressor LexA | 81 (100) | TT**AGA**TTTTGGATTGA**TCT**GT TG**TGT**CACCTGACG**ACA**AA | 88 5 | CTTCATAAT | 72 |
| *all5039* (*atpB*) | WP_250121196.1 | F0F1 ATP synthase subunit beta | 88 (100) | TA**AGT**CTGCAGTAAT**ACT**GT | 346 | TTGTAAATT AGTTAGAAT GGATATGAT | 55 370 705 |

^a^ as computed by a two proteins BLASTP analysis

^b^ putative ChroLexA-boxes were searched in intergenic regions by using the previously reported criteria (Srivastava, et al. 2022):

i) Include all sequences with the AGT-N4-11-ACT motif and, if this search didn't give any result,

ii) Include sequences having AG(T/a/c/g) and tGT in the left arm and AC(T/a), (t/g)CT, A(a/t)T in the right arm of the previous motif. In this latter case, only motifs with a perfect palindrome of at least 4 bases were included

^c^ distance between the last nucleotide of the motif right arm and the gene start codon

^d^ Promoters were identified using the BPROM web-service (<http://www.softberry.com/berry.phtml?topic=bprom&group=programs&subgroup=gfindb>)

^e^ distance between the last nucleotide of the predicted promoter -10 region and the gene start codon
